# Clinical Society of Maryland

**Published:** 1894-07

**Authors:** H. O. Reik

**Affiliations:** Secretary; 525 N. Howard Street; Baltimore


### Society Reports.

### CLINICAL SOCIETY OF MARYLAND.

BALTIMORE, May 18, 1894.

The 297th regular meeting of the Clinical Society was called to order by DR. GEORGE J. PRESTON. President, *pro tem.*

DR. C. W. MITCHELL read a paper on "Rickets." The belief that the importance of rickets is generally underestimated by American physicians has led me to bring this subject before the Society. Errors widely prevail as to the frequency of its occurrence, and the direct bearing it has upon the course of many diseases of children. While it prevails most widely in the densely populated cities of Europe, it is yearly becoming more frequent here.

Numerous theories have been offered as to its etiology, and while there is probably some truth in each of them, they teach us on the whole that there is probably nothing specific in the nature of rickets, that it is a perversion of the normal metabolism of the body, and that any cause of general malnutrition may give rise to it. Probably the most common cause of it is improper feeding, and I should like to emphasize the fact that it occurs among rich as well as poor children. If looked for with care, it will be found to affect the child of the millionaire as well as the bow-legged negro of the back alley.

Unfortunately riches will not provide mother's milk. The breast-fed offspring of the poor woman is less apt to suffer from rickets than the artificially fed child of the society woman. Of course I do not mean to state that the gross deformities are so frequently found among the children of the rich, because when they begin to show themselves they are properly treated, but I do believe that changes in the chest wall, which are the most constant and dangerous of all the signs of rickets, are frequently overlooked among the children of the well-to-do.

Whenever the changes in the chest wall are marked they lessen the breathing force of the patient to so great an extent as to cause simple catarrhal inflammations of the respiratory organs, to be attended by grave dangers. So, in cases so mild as to be overlooked unless the child be stripped, there is apt to occur dangerous complications on the part of the lungs, in the course of disease. which in perfectly healthy children need excite no anxiety.

In rachitic children simple bronchitis is attended by danger, because the respiratory movements being weakened the course of the disease is prolonged and broncho-pneumonia results. So, too, in measles, whooping-cough, etc., the presence of rachitic changes renders the prognosis always uncertain. Most text-books state that rickets occur most commonly from the sixth to eighteenth month, but recent investigations show that many cases begin earlier.

As deformity of the chest is the earliest of all signs, we should have the child, in all cases of bad nutrition, stripped from time to time and carefully examine all the bony structures.

Care should be had not to keep the child upon any one article of food to the exclusion of others for too long. The greatest care and attention should be given to the food. When rickets actually occurs the child should be kept in the sunlight as much as possible. Phosphorus, cod-liver oil, iron and arsenic are of value medicinally, and Dr. Abel, of Johns Hopkins University, has recently demonstrated that lime-water, hitherto thought to be inert, may exercise great influence on animal metabolism.

In conclusion I wish to empasize the following points :

1. Deformity of the chest is the earliest, as well as the most formidable manifestation of the disease.

2. Rachitis is probably not a specific disease, but results from any of the causes of malnutrition.

3. It is not only preventable, but may be promptly arrested when early recognized.

4. Be on the lookout for rickets in private as well as in dispensary practice.

DR. WM. OSLER said he was always interested in a discussion of rickets, for it was very remarkable how often common cases of this disease were overlooked by physicians. It is certainly increasing in this country.

H. O. REIK, M.D., Secretary.

525 *N. Howard Street.*

